# Genome-Wide Analysis of *leafbladeless1*-Regulated and Phased Small RNAs Underscores the Importance of the *TAS3* ta-siRNA Pathway to Maize Development

**DOI:** 10.1371/journal.pgen.1004826

**Published:** 2014-12-11

**Authors:** Marcela C. Dotto, Katherine A. Petsch, Milo J. Aukerman, Mary Beatty, Molly Hammell, Marja C. P. Timmermans

**Affiliations:** 1 Cold Spring Harbor Laboratory, Cold Spring Harbor, New York, United States of America; 2 DuPont Crop Genetics, Wilmington, Delaware, United States of America; 3 Pioneer-DuPont, Johnston, Iowa, United States of America; Harvard University, United States of America

## Abstract

Maize *leafbladeless1* (*lbl1*) encodes a key component in the trans-acting short-interfering RNA (ta-siRNA) biogenesis pathway. Correlated with a great diversity in ta-siRNAs and the targets they regulate, the phenotypes conditioned by mutants perturbing this small RNA pathway vary extensively across species. Mutations in *lbl1* result in severe developmental defects, giving rise to plants with radial, abaxialized leaves. To investigate the basis for this phenotype, we compared the small RNA content between wild-type and *lbl1* seedling apices. We show that LBL1 affects the accumulation of small RNAs in all major classes, and reveal unexpected crosstalk between ta-siRNA biogenesis and other small RNA pathways regulating transposons. Interestingly, in contrast to data from other plant species, we found no evidence for the existence of phased siRNAs generated via the one-hit model. Our analysis identified nine *TAS* loci, all belonging to the conserved *TAS3* family. Information from RNA deep sequencing and PARE analyses identified the tasiR-ARFs as the major functional ta-siRNAs in the maize vegetative apex where they regulate expression of *AUXIN RESPONSE FACTOR3* (*ARF3*) homologs. Plants expressing a tasiR-ARF insensitive *arf3a* transgene recapitulate the phenotype of *lbl1*, providing direct evidence that deregulation of ARF3 transcription factors underlies the developmental defects of maize ta-siRNA biogenesis mutants. The phenotypes of *Arabidopsis* and *Medicago* ta-siRNA mutants, while strikingly different, likewise result from misexpression of the tasiR-ARF target *ARF3*. Our data indicate that diversity in *TAS* pathways and their targets cannot fully account for the phenotypic differences conditioned by ta-siRNA biogenesis mutants across plant species. Instead, we propose that divergence in the gene networks downstream of the ARF3 transcription factors or the spatiotemporal pattern during leaf development in which these proteins act constitute key factors underlying the distinct contributions of the ta-siRNA pathway to development in maize, *Arabidopsis*, and possibly other plant species as well.

## Author Summary

Mutations in maize *leafbladeless1* (*lbl1*) that disrupt ta-siRNA biogenesis give rise to plants with thread-like leaves that have lost top/bottom polarity. We used genomic approaches to identify *lbl1*-dependent small RNAs and their targets to determine the basis for these polarity defects. This revealed substantial diversity in small RNA pathways across plant species and identified unexpected roles for LBL1 in the regulation of repetitive elements within the maize genome. We further show that only ta-siRNA loci belonging to the *TAS3* family function in the maize vegetative apex. The *TAS3*-derived tasiR-ARFs are the main ta-siRNA active in the apex, and misregulation of their *ARF3* targets emerges as the basis for the *lbl1* leaf polarity defects. Supporting this, we show that plants expressing *arf3a* transcripts insensitive to tasiR-ARF-directed cleavage recapitulate the phenotypes observed in *lbl1*. The *TAS3* ta-siRNA pathway, including the regulation of *ARF3* genes, is conserved throughout land plant evolution, yet the phenotypes of plants defective for ta-siRNA biogenesis are strikingly different. Our data leads us to propose that divergence in the processes regulated by the ARF3 transcription factors or the spatiotemporal pattern during development in which these proteins act, underlies the diverse developmental contributions of this small RNA pathway across plants.

## Introduction

Small RNAs are important regulators of development, particularly in plants, where many of the abundant and conserved microRNAs (miRNAs) target transcription factors that direct or reinforce cell fate decisions [1]. Consequently, mutations in genes required for miRNA processing or function condition defined developmental defects. Likewise, plants defective for the biogenesis of trans-acting short interfering RNAs (ta-siRNAs) show distinctive patterning defects due to the deregulation of key developmental targets [1]. ta-siRNAs are generated in response to miRNA activity via one of two possible mechanisms, referred to as the “one-hit” and “two-hit” pathways. In both pathways, a single miRNA-guided cleavage event triggers the conversion of target transcripts into long double stranded RNAs by RNA-DEPENDENT RNA POLYMERASE6 (RDR6) and SUPPRESSOR OF GENE SILENCING3 (SGS3), and sets the register for the subsequent production of phased 21-nt siRNAs by DICER-LIKE4 (DCL4) [2]-[4]. In the one-hit pathway, transcripts targeted by a single, typically 22-nt, miRNA will generate ta-siRNAs downstream of the miRNA cleavage site, whereas transcripts producing ta-siRNAs via the two-hit pathway harbor two binding sites for 21-nt miRNAs and the ta-siRNAs are processed upstream of the cleaved 3′ miRNA target site [5]–7. Analogous to miRNAs, a subset of the phased ta-siRNAs act at the post-transcriptional level to repress the expression of genes involved in development or other cellular processes.

The phenotypes conditioned by mutations affecting ta-siRNA biogenesis vary greatly across species. In *Arabidopsis,* such mutants exhibit a relatively subtle phenotype, developing downward curled leaves that are weakly abaxialized and undergo an accelerated transition from the juvenile to the adult phase [2]–[3]. These defects result from misregulation of the AUXIN RESPONSE FACTOR ARF3, which is targeted by *TAS3*-derived ta-siRNAs, termed tasiR-ARFs [8]–[10]. Biogenesis of the *TAS3* ta-siRNAs follows the two-hit model and involves a subspecialized pathway, which requires the unique association of miR390 with its effector AGO7 to trigger siRNA production [11]. Localized expression of *TAS3* and *AGO7* confines tasiR-ARF biogenesis to the adaxial/upper most cell layers of developing leaves, which then limits accumulation of ARF3 to the abaxial/lower side [12]. The developmental defects of *sgs3, rdr6*, and *dcl4* are phenocopied by mutations in *AGO7* and *TAS3A*, as well as by expression of tasiR-ARF- insensitive *ARF3* transgenes [2], [8]–[9], [13], indicating that the contribution of ta-siRNAs to *Arabidopsis* development is primarily mediated by tasiR-ARFs.

**Figure 1:**
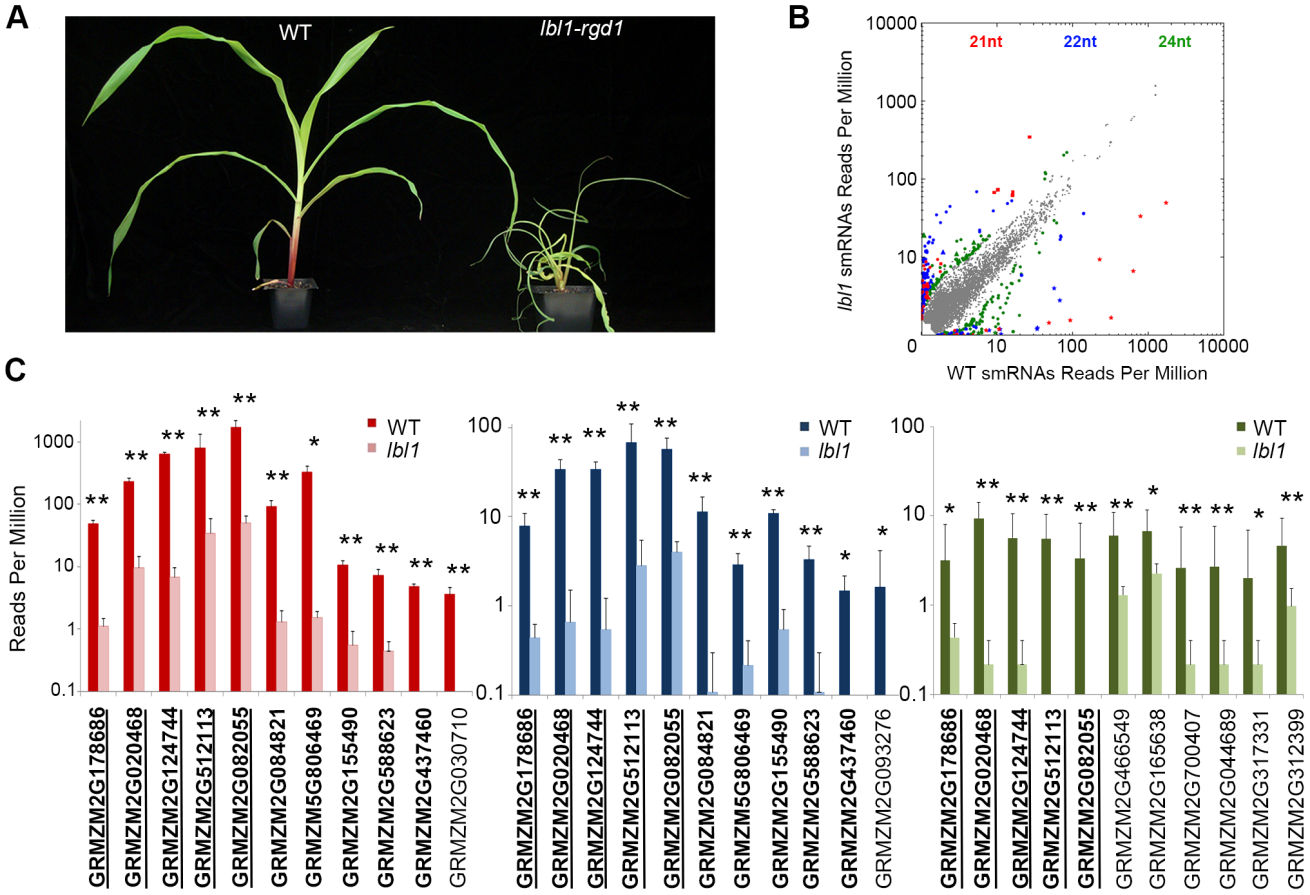
*lbl1* affects small RNA biogenesis at select loci. (A) Compared to wild-type, *lbl1-rgd1* mutant seedlings show a reduced stature and develop radial, abaxialized leaves. (B) *lbl1* has a relatively subtle effect on the overall small RNA population; in three independent biological replicates 79, 172, and 209 loci generate 21-, 22-, or 24-nt small RNAs, respectively, that are significantly changed (q-value<0.05) at least 2-fold between wild-type and *lbl1*. Grey dots mark the relative normalized abundance of small RNAs not significantly changed between wild-type and *lbl1*. Colored dots correspond to significantly changed small RNAs: red, 21-nt; blue, 22-nt; green, 24-nt. (C) Low copy genic regions accumulating significantly fewer 21-nt (left), 22-nt (middle) and/or 24-nt (right) siRNAs in *lbl1*. Genes marked in bold and underlined show reduced levels for all three siRNA classes. Genes marked in bold show reduced 21- and 22-nt siRNAs. Note that the abundance of 21-nt siRNAs at these loci is generally substantially higher. Values are reported on a log scale and represent the mean normalized read counts (RPM) and standard deviation across three independent biological replicates. * adjusted p-value<0.05; ** adjusted p-value<0.01.

In contrast to *Arabidopsis*, loss of AGO7 activity in *Medicago* results in the formation of highly lobed leaves [14], and mutants defective for ta-siRNA biogenesis components in rice and tomato exhibit severe defects in meristem maintenance, mediolateral blade expansion, and adaxial-abaxial leaf polarity [15],[16]. Likewise, mutations in maize *lbl1* and *ragged seedling2* (*rgd2),* which encode the orthologs of SGS3 and AGO7, respectively, have severe effects on meristem function and leaf development [17]–[18]. *lbl1* mutants, in particular, develop radial, fully abaxialized leaves (Fig. 1A).

Importantly, while the *TAS3* ta-siRNA pathway is evolutionarily conserved, the number and nature of phased siRNA loci vary greatly between plant species [19]. In *Arabidopsis*, three *TAS* families have been described in addition to *TAS3*. *TAS1*-, *TAS2*-, and *TAS4*-derived ta-siRNAs are generated via the one-hit model following miR173- or miR828-directed cleavage, and function in the regulation of members in the MYB transcription factor and PPR families [4], [20]–[21]. Each of these pathways has been identified in other plant species, but their evolutionary origin appears to lie within the eudicots [19]. Depending on the species, variation is seen in the genes targeted by the ta-siRNAs derived from these *TAS* loci, as well as in the miRNA that triggers their biogenesis [22], [23]. In addition, apparent species-specific *TAS* pathways may exist, as novel *TAS* loci with unique targets have been identified in tomato and the moss *Physcomitrella patents* [24], [25]. Moreover, genome-wide small RNA analyses in a number of plant species have uncovered clusters of phased siRNAs, generated primarily via the one-hit model that, unlike the ta-siRNAs, are proposed to act in *cis*. These are generally referred to as phasiRNAs and are processed from non-coding transcripts, such as in the panicles of the grasses [26]–[28], or from protein-coding genes, including members of the NB-LRR, MYB, and PPR gene families [19]. The set of genes regulated by phased siRNAs thus varies widely across plant species.

The basis for the phenotypes of maize ta-siRNA biogenesis mutants remains unclear. In light of the tremendous diversity in phased siRNAs seen across plant species, it is conceivable that *TAS* loci, other than the four known *TAS3* genes [17], exist in maize. Moreover, phased siRNAs other than the tasiR-ARFs may target genes with roles in development, and contribute to the defects seen in *lbl1* mutants. To assess these possibilities and to obtain a comprehensive view of LBL1-dependent siRNAs active in the maize vegetative apex, where the mutant phenotype manifests itself, we compared the small RNA content between wild-type and *lbl1* shoot apices. This revealed unexpected contributions of LBL1 to the regulation of transposons, particularly the *gyma* class of LTR-retrotransposons. Interestingly, in contrast to other plant species, we found no evidence for the existence of phased siRNAs generated via the one-hit model. Our analyses identified nine *TAS* loci all belonging to the *TAS3* family. Data from RNA deep sequencing and PARE analysis present the *ARF3* genes as the only LBL1-dependent small RNA targets with a role in development. Consistent with this finding, plants expressing a tasiR-ARF insensitive *arf3a* transgene recapitulate the phenotype of *lbl1* mutants. These findings underscore the importance of the tasiR-ARF - ARF3 regulatory module to maize development, and indicate that diversity in *TAS* pathways and their targets cannot fully account for the phenotypic differences conditioned by ta-siRNA biogenesis mutants across plant species. Instead, divergence in the gene networks downstream of the ARF3 transcription factors or the spatiotemporal pattern in which these tasiR-ARF targets act emerge as a testable hypotheses to explain the diverse contributions of the ta-siRNA pathway to development in maize, *Arabidopsis*, and possibly other plant species as well.

## Results/Discussion

### LBL1 affects 21-, 22-, and 24-nt small RNA biogenesis at select loci

To understand the basis for the *lbl1* phenotype, we compared the small RNA content between vegetative apices, comprising the shoot apical meristem (SAM) and up to five leaf primordia, of two-week old B73 and *lbl1-rgd1* seedlings. Three independent biological replicates were analyzed for each genotype. Approximately 92% of the 18- to 26-nt reads in both sets of libraries mapped to the unmasked B73 reference genome (S1 Table). Of the mapped reads, 43–48%, corresponded to unique small RNAs suggesting that, despite the highly repetitive nature of the maize genome, close to half of the small RNAs expressed in the vegetative apex are distinct. The small RNA size distribution profiles in both wild-type and *lbl1* resemble those described previously for maize [29]–[30], suggesting that LBL1, in contrast to components of the heterochromatic siRNA pathway [29], has a relatively subtle effect on the overall small RNA population (S1 Figure). However, consistent with a role for SGS3 proteins in the biogenesis of 21-nt secondary siRNAs [2], [4], the 21-nt small RNA population is slightly reduced in *lbl1* compared to wild-type. In addition, an unexpected modest reduction in the 22- and 24-nt small RNA fractions is seen in *lbl1* (S1 Figure).

To more precisely define the effects of LBL1 on small RNA biogenesis, we identified genomic loci that differentially accumulate 21-, 22-, or 24-nt small RNAs in wild-type versus *lbl1* apices (S2 Figure). Consistent with a relatively subtle effect of *lbl1* on the overall small RNA population, this identified 79, 172, and 209 loci that generate 21-, 22-, or 24-nt small RNAs, respectively, that are significantly changed (q-value<0.05) at least 2-fold between wild-type and *lbl1* (Fig. 1B; S1 Dataset). A small subset of these (11/79), correspond to low copy genic regions that generate significantly fewer 21-nt small RNAs in *lbl1*, properties predicted for phasiRNA and ta-siRNA loci (Fig. 1C; S1 Dataset). Indeed, the four previously described *TAS3* genes, *tas3a*-*d* [17], are among these loci. Five additional genes appear non-coding, whereas the remaining two correspond to *arf3a* and *arf3d* (Fig. 1C; S1 Dataset). With the exception of *arf3a*, each of these loci also generate 22-nt LBL1-dependent siRNAs and five differentially accumulate 24-nt small RNAs, albeit generally to substantially lower levels than seen for the corresponding 21-nt small RNAs (Fig. 1C). One additional gene (GRMZM2G093276), encoding a zinc/iron transporter, also accumulates 22-nt small RNAs that are significantly reduced in *lbl1*. Finally, six predicted protein-coding genes of unknown function generate low levels of 24-nt small RNAs that are lost or significantly reduced upon mutation of *lbl1*. Thus, a total of 18 distinct low copy genic regions generate small RNAs in an apparent LBL1-dependent manner. These include the four known maize *TAS* loci, *tas3a-d*, and the remaining present candidate novel phasiRNA or *TAS* loci that are active in the vegetative apex and may contribute to the developmental defects resulting from mutation of *lbl1*.

### Biogenesis of phased siRNAs in the vegetative apex

To further discern whether additional phased siRNA loci are active in the vegetative maize apex, we developed a pipeline that scans the genome for clusters of siRNAs showing a regular phasing of 21, 22, or 24 nucleotides (S2 Figure). This pipeline includes a phasing score calculation (P-score) that identifies clusters in which the majority of small RNAs produced are phased. With a P-score threshold (P≥25) that has been shown to identify 7 of the 8 *TAS* loci in *Arabidopsis* [21], [31], we identified 16 phased 21-nt siRNA clusters, 102 phased 22-nt siRNA clusters, and 8 phased 24-nt siRNA clusters (S2 Dataset). However, combining this analysis with the differential small RNA accumulation data described above (S1 Dataset), showed that small RNA levels in most of the clusters are not changed significantly between wild-type and *lbl1*. In fact, small RNA accumulation at just 8 of the phased siRNA clusters is changed in the *lbl1* mutant (S3A Figure). A closer inspection of the remaining clusters indicates that these correspond primarily to repetitive regions in the genome. Moreover, these siRNA clusters are typically embedded within large windows, frequently spanning over 5 kb, that generate an uncharacteristically high number of small RNA reads, which inflates the P-score (see Materials and Methods). As such, it is unlikely that these clusters correspond to new *TAS* loci or other miRNA-triggered phased siRNA loci. Instead these loci, particularly the LBL1-independent 22-nt siRNAs, appear to be processed from long hairpin RNAs or overlapping antisense transcripts (S3B-C Figure). In *Arabidopsis*, natural antisense transcripts are processed by DCL1 into 21-nt small RNAs [32], [33], whereas long hairpin RNAs are targeted by multiple DCL enzymes to give rise to variably sized small RNAs [34]. The preferential processing of such double stranded RNAs into 22-nt siRNAs presents a possible basis for the uncharacteristically high overall abundance of 22-nt small RNAs in maize (S1 Figure), and suggests diversification in the action of DCL family members between *Arabidopsis* and maize.

Of the eight phased siRNA clusters whose small RNA levels are significantly changed in *lbl1*, one generates 24-nt phased siRNAs (S3A Figure; S2 Dataset). However, its small RNA levels are increased in *lbl1*, again making it unlikely that this cluster corresponds to a novel phased secondary siRNA locus. Thus, in the vegetative apex, only seven loci generate phased siRNAs with a P-score≥25 in an LBL1-dependent manner. These phased siRNAs are 21-nt in size and are derived from low copy genic regions predicted to generate non-coding transcripts. Importantly, the four previously described maize *TAS3* loci, *tas3a-d* [17], are among these seven loci (S2 Dataset). The three additional loci, GRMZM5G806469, GRMZM2G082055, and GRMZM2G512113, present novel maize phased siRNA loci that could contribute to the developmental defects resulting from mutation of *lbl1*.

### ta-siRNA biogenesis in the maize vegetative apex is miR390-dependent

**Figure 2:**
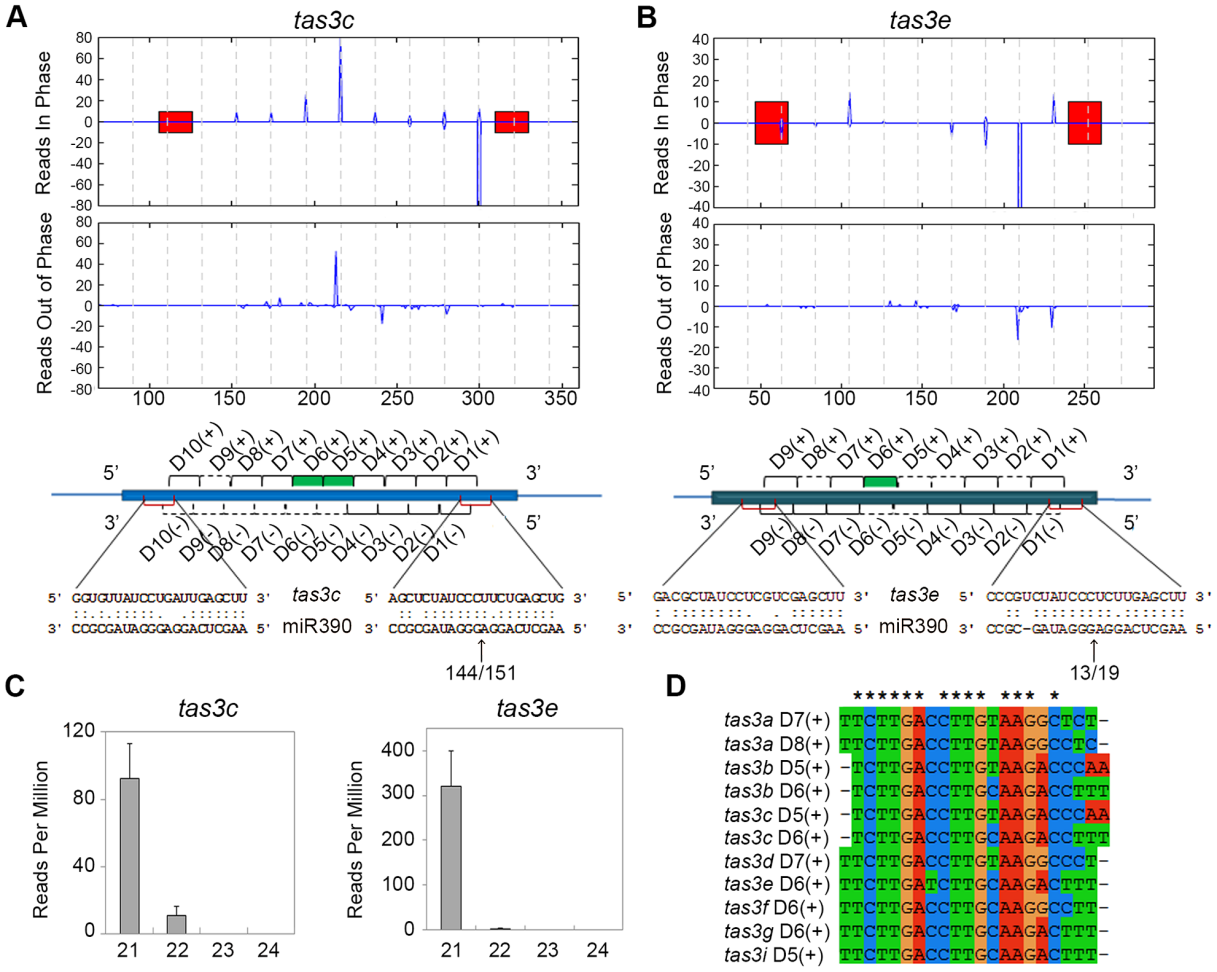
Organization of *TAS3* loci generating phased 21-nt ta-siRNAs. (A, B) Normalized read counts (RPM) for ta-siRNAs in phase with the 3′ miR390 cleavage site (top graphs) and for out of phase small RNAs (bottom graphs) are shown for *tas3c* (A) and *tas3e* (B). Red bars, miR390 binding sites; vertical dashed lines, the 21-nt register. Annotations of the *TAS3* precursors are shown beneath, with alignments of miR390 to the *tas3c* and *tas3e* precursors and the number of PARE signatures in the small window (W_S_) over the large window (W_L_) at the 3′ miR390 target sites indicated. Black brackets; ta-siRNAs detected in the vegetative apex libraries; dashed lines, predicted ta-siRNAs not detected in our libraries; red brackets, miR390 binding sites; green bars, tasiR-ARFs. (C) Size distribution profiles for small RNAs derived from *tas3c* and *tas3e* showing most are 21-nt long. Values shown are the mean normalized read counts (RPM) and SD from three independent biological replicates. (D) Alignment of the eleven tasiR-ARFs processed from the maize *TAS3* loci. Asterisks indicate 100% identity.

A closer analysis of the three novel phased siRNA loci indicates that these represent new members of the *TAS3* family. Transcripts from these loci contain two miR390 binding sites and have the potential to generate small RNAs homologous to tasiR-ARFs (Fig. 2A-D; S4A-G Figure). As mentioned above, two additional low copy regions in the genome (GRMZM2G155490 and GRMZM2G588623) generate 21-nt LBL1-dependent small RNAs from predicted non-coding transcripts (Fig. 1C; S1 Dataset). While both loci did not pass the P-score filter in the phased siRNA analysis (S2 Dataset), a closer analysis indicates that both contain two miR390 binding sites (S4D, F Figure). Both loci generate relatively few small RNAs in vegetative apices with many being out of phase (S4 D, F Figure), presenting a likely explanation for the observed low P-score. Supporting this, a similar P-score analysis of 21-nt small RNAs in *Arabidopsis* failed to detect the confirmed *TAS4* ta-siRNA locus due to the low abundance of its reads [21]. Data from PARE (parallel analysis of RNA ends; [35]) libraries generated from B73 apices, which allow the detection of small RNA-directed mRNA cleavage products, confirms that the 3′ miR390 target site in transcripts generated from all nine loci is cleaved (Fig. 2A-B). As such, the new loci were named *tas3e* to *tas3i* (Table 1).

**Table 1:** Maize *TAS* loci.

Name	Gene ID	Number of ta-siRNAs *	Number of tasiR-ARFs	Phasing score	Number of ta-siRNA reads per locus †	Fold change (WT/*lbl1*) ‡
*tas3a*	GRMZM2G178686	26	2	63.70	408	52.9
*tas3b*	GRMZM2G020468	20	2	41.46	1894	28.15
*tas3c*	GRMZM2G084821	20	2	30.54	854	84.58
*tas3d*	GRMZM2G124744	24	1	42.81	6382	110.92
*tas3e*	GRMZM5G806469	18	1	50.08	3475	251.64
*tas3f*	GRMZM2G155490	28	1	11.80	22	23.2
*tas3g*	GRMZM2G082055	20	1	50.57	18325	40.71
*tas3h*	GRMZM2G588623	20	0	4.97	16	19.25
*tas3i*	GRMZM2G512113	18	1	45.31	8013	28.59

*Number of predicted ta-siRNAs between miR390 binding sites from both strands.

† Total number of reads in WT samples.

‡ Adjusted p-value<0.05.

The number of potential ta-siRNAs generated from these loci varies from 9 to 13 per strand (Table 1). However, not all predicted ta-siRNAs are detected in the vegetative apex; in fact, only 99 of the 194 possible ta-siRNAs are present in our datasets (Fig. 2A-D; S4A-G Figure). Moreover, our analyses reveal that while not all ta-siRNAs are 21-nt in size, only the 21-nt long small RNAs are phased, and this class is more abundant than the longer, or out of phase, small RNAs (Fig. 2C; S4A-G Figure). This indicates that DCL4 processing is occasionally out of phase and/or a different DCL enzyme takes over. Intriguingly, besides the generally low ta-siRNA levels coming from *tas3f* and *tas3h*, the potential *tas3f*-derived tasiR-ARF was not detected in our small RNA libraries, and *tas3h* lacks the potential to generate this small RNA altogether. Although it is conceivable that *tas3f* and *tas3h* have other biological roles, it seems that, with respect to the vegetative apex, these loci are diverging and losing their function in the tasiR-ARF pathway.

A similar analysis of the remaining seven low copy regions accumulating significantly fewer 22- and 24-nt small RNAs in *lbl1* (Fig. 1C; S1 Dataset), make it unlikely that these represent new phased siRNA loci. Target prediction and PARE analysis indicate that none of these genes are targeted by the known maize miRNAs or ta-siRNAs (see below). Interestingly, similar analyses of phased siRNA clusters in rice and *Brachypodium* inflorescences did detect 24-nt phased siRNA loci in addition to 21-nt phased siRNAs [26]–[28]. miR2275, which triggers the biogenesis of the rice and *Brachypodium* 24-nt phased siRNAs, is not detected in the maize vegetative apex. This miRNA is, however, present in maize inflorescence tissues [26], implying that some of the observed diversity may reflect tissue-specificity of small RNA pathways. Likewise, many of the 21-nt phased siRNAs identified in rice and *Brachypodium* inflorescences form a family distinct from the *TAS3* loci whose biogenesis is triggered by miR2118, which in maize accumulates specifically in the inflorescences [26].

In addition, no loci generating phased siRNAs via the “one-hit” model were identified in our analysis, despite the presence of 22-nt small RNAs shown to serve as triggers in this process [6], [7]. The maize apex appears unique in this regard, as all similar genome-wide analyses of small RNAs in seedling tissues in other species identified both types of phased siRNA loci [21], [31], [36], [37]. Perhaps such miRNAs are not loaded into maize AGO proteins or, alternatively, loading of these miRNAs fails to trigger a reprogramming of the RNA-induced silencing complex required to trigger secondary siRNA biogenesis [7]. Taken together, the above data indicate that, in the maize vegetative apex, phased secondary siRNAs are generated from nine loci, all belonging to the *TAS3* family. This rules out a possible contribution of novel *TAS* families to the distinctive phenotype of *lbl1* mutants.

### *ARF3* genes are primary targets of ta-siRNAs in the maize shoot apex

**Figure 3:**
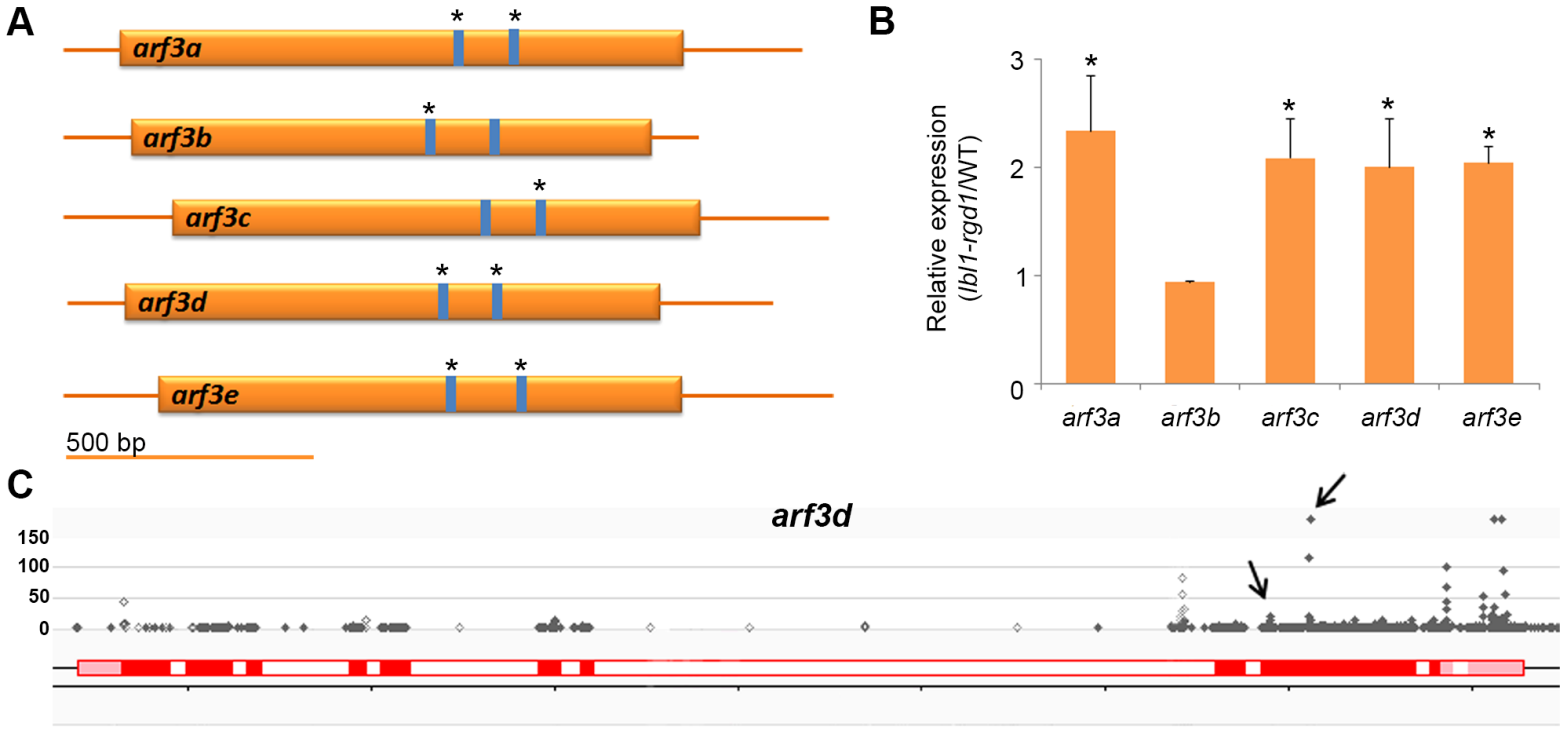
Regulation of *ARF3* genes by ta-siRNAs in the maize shoot apex. (A) Transcripts of the five maize *ARF3* genes each contain two tasiR-ARF target sites spaced approximately 200 bp apart. PARE analysis indicates that both sites in *arf3a*, *arf3d* and *arf3e* transcripts are cleaved, whereas only one target site was validated for the *arf3b* and *arf3c* transcripts (asterisks). (B) Expression for four of the five *ARF3* genes is significantly changed between wild-type and *lbl1* in RNAseq and qRT-PCR analyses. Transcript levels determined by qRT-PCR (mean ± SD; n = 3) normalized to wild-type are shown (* p<0.05). The RPKM values for the *ARF3* genes in wild-type and mutant samples are reported in S3 Dataset. (C) The distribution of PARE signatures at *arf3d*, showing the presence of high abundance signatures at the predicted tasiR-ARF target sites (arrows). Grey dots, PARE signatures and their abundance in reads per 15 million reads; red boxes, exons; white boxes, introns; light red boxes, UTRs.

Considering that the *lbl1* phenotype is not explained by the presence of novel *TAS* loci, we next asked whether *TAS3*-derived ta-siRNAs other than the tasiR-ARFs contribute to the developmental defects seen in *lbl1*. To test this possibility, we constructed degradome libraries from B73 apices, and used these in target prediction [4] and PARE analyses to identify potential targets for all 99 *TAS3*-derived ta-siRNAs detected in our small RNA libraries. Allowing for a maximum score of 4.5, well above the maximum score of 3.0 obtained for the tasiR-ARF *ARF3* duplexes, and filters comparable to Zhai et al. [31], PARE analysis confirmed 18 cleavage sites in 11 target genes (S3 Dataset). Five of the *TAS3* transcripts are among the verified tasiRNA targets, indicating possible feedback regulation in the tasiRNA pathway. The remaining targets include GRMZM2G018189, which encodes an uncharacterized protein homologous to AtSLT1 that mediates salt tolerance in yeast [38], as well as the five members of the maize *ARF3* gene family (Fig. 3A-C; Table 2). The *ARF3* genes are targeted by multiple closely related tasiR-ARFs, and account for 8 of the validated cleavage sites (Figs. 2D; 3A; S3 Dataset).

**Table 2:** Validation of predicted targets.

Target	Annotation	Fold change (*lbl1*/WT) *	Significant †	ta-siRNA	Score	PARE ‡
GRMZM2G030710	*arf3a*	2.12	Yes	tasiR-ARFs	0 to 3	YES
GRMZM2G441325	*arf3b*	1.23	No	tasiR-ARFs	1.5 to 3	YES
GRMZM2G056120	*arf3c*	2.56	Yes	tasiR-ARFs	0.5 to 2.5	YES
GRMZM2G437460	*arf3d*	3.05	Yes	tasiR-ARFs	0 to 3	YES
GRMZM5G874163	*arf3e*	2.69	Yes	tasiR-ARFs	0.5 to 3	YES
GRMZM2G018189	*slt1*	0.99	No	gD2 (-)	3.5	YES

*RPKM *lbl1*/RPKM B73.

† Differential expression (RNA seq) adjusted p-values<0.05.

‡ Predicted cleavage site validated in PARE analysis.

To assess a possible contribution of the ta-siRNA targets to the *lbl1* seedling defects, we next determined whether their expression levels are changed in the mutant. The same tissue samples from wild-type and *lbl1* vegetative apices were used to identify differentially expressed genes by RNA deep sequencing (S2 Table; S3 Dataset). 1116 genes show differential expression in *lbl1* compared to wild-type (fold change ≥2, q-value<0.05). Of the verified ta-siRNA targets, transcript levels for *arf3a* and *arf3c-e* are significantly increased in *lbl1* (Table 2). Misregulation of these abaxial determinants is consistent with the *lbl1* leaf polarity defects [17], but, unexpectedly, expression for *arf3b* is unchanged in the mutant. qRT-PCR analysis of RNA isolated from *lbl1-rgd1* apices similarly shows that transcript levels for *arf3a* and *arf3c-e* are significantly increased, by approximately 2-fold, whereas expression of *arf3b* remains unchanged (Fig. 3B). The latter is correlated with a relatively low number of PARE signatures at *arf3b* (S3 Dataset), and suggests that this *arf3* member may not substantially add to the adaxial-abaxial polarity defects seen in *lbl1*. Likewise, transcript levels for GRMZM2G018189 are not significantly changed in the mutant, and the number of PARE signatures precisely at the predicted cleavage site is low. Along with its predicted role in salt detoxification, this makes a contribution of GRMZM2G018189 to the *lbl1* phenotype unlikely.

Interestingly, *arf3a* and *arf3d* generate 21-nt small RNAs, which are significantly downregulated in *lbl1* (Fig. 1C; S1 Dataset). Both *ARF3* genes contain two tasiR-ARF target sites and the majority of the LBL1-dependent siRNAs map between these sites (Fig. 3A; S3 Dataset). This suggests that tasiR-ARF-mediated regulation of *arf3a* and *arf3d* triggers the biogenesis of third tier secondary siRNAs also via the two-hit model, and implies positive feedback in the regulation of *ARF3* expression. Although the significance of such feedback regulation remains to be established, considering the role of tasiR-ARFs in limiting the activity of ARF3 abaxial determinants to the lower side of leaf primordia, positive feedback could be important to reinforce adaxial cell fate, and to sharpen or maintain the boundary between adaxial and abaxial domains [10], [17].

In contrast to dicot species [21], [31], [36], [37], the miR390-dependent *TAS3* ta-siRNA pathway, thus, is the only phased secondary siRNA pathway active in the maize shoot apex. This ta-siRNA pathway, including the regulation of ARF targets, is conserved throughout land plant evolution [4], [5], [10]–[17], although substantial diversity has accumulated over evolutionary time. The tasiR-ARFs show sequence divergence, and not all maize *TAS3* genes generate this biologically active ta-siRNA (Fig. 2D; S4F Figure). In addition, like the *Physcomitrella patens AP2* targets [24], GRMZM2G018189 presents a possible novel maize *TAS3* ta-siRNA target. Despite such divergence in the *TAS3* pathway, the *ARF3* genes form the prime ta-siRNA targets also in maize, suggesting that the developmental phenotype of *lbl1* results, at least in part, from a failure to correctly regulate their expression.

### Other LBL1-dependent small RNAs contribute negligibly to development

**Figure 4:**
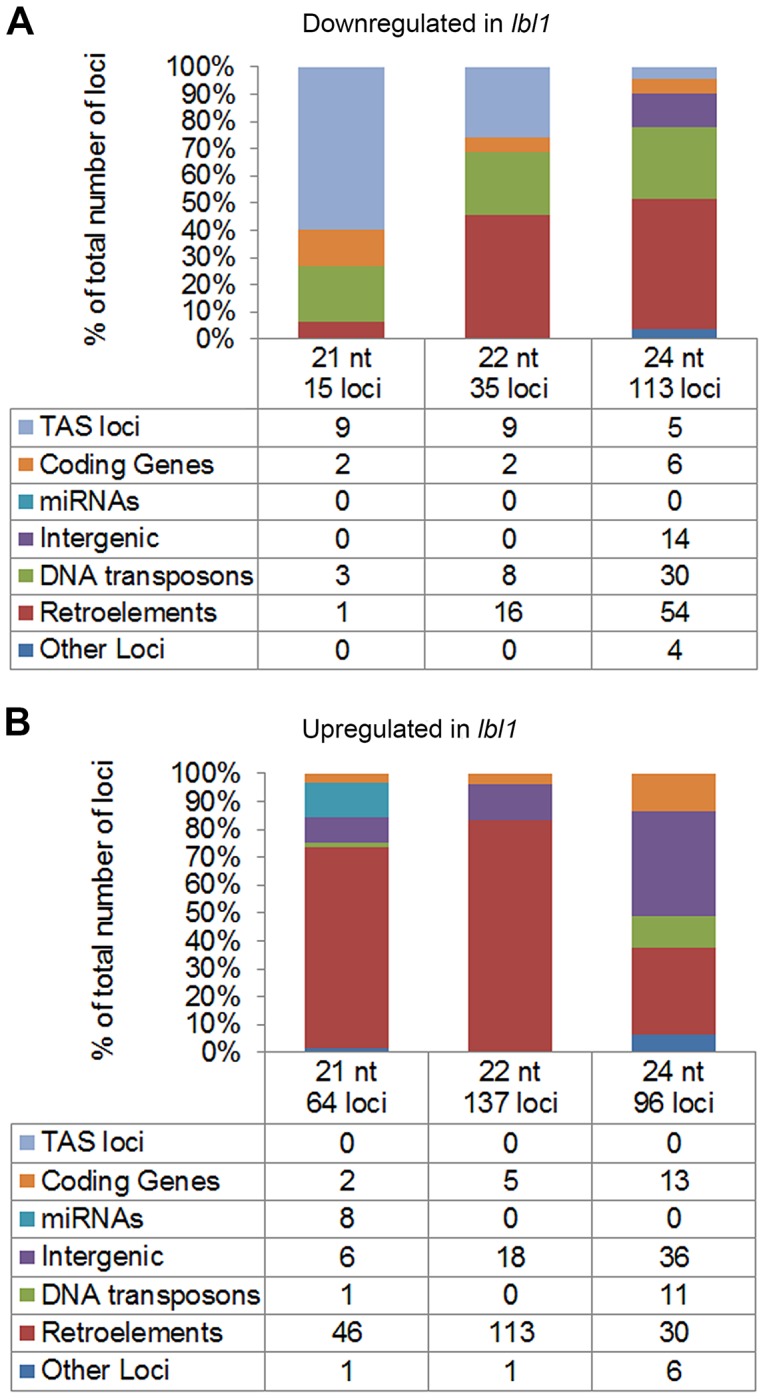
LBL1 affects the accumulation of 21-, 22-, and 24-nt small RNAs. (A) Annotation of loci that accumulate significantly fewer small RNAs in *lbl1* than wild-type. (B) Annotation of loci that accumulate significantly more small RNAs in *lbl1* compared to wild-type. Tables list the number of loci in each category and bar graphs show their relative frequencies.

A requirement for LBL1 in the production of phased siRNAs explains, however, only a subset of the small RNA level changes identified in the vegetative apex of *lbl1* mutants. A further 68, 161, and 198 loci generating 21-, 22-, or 24-nt small RNAs, respectively, show a significant change (q-value<0.05) in small RNA accumulation of at least 2-fold between wild-type and *lbl1* apices (S1 Dataset, Fig. 4A-B), and these could conceivably contribute to the developmental defects of *lbl1* mutants. To assess this possibility and to gain insight into the possible function of these LBL1-dependent siRNAs, we first determined whether genes differentially accumulating small RNAs in *lbl1* show a corresponding change in transcript levels. In addition to the *ARF3* and *TAS3* loci discussed above, 23 protein coding genes within the maize filtered and working gene sets show a significant difference in 21-, 22-, or 24-nt small RNA accumulation between wild-type and *lbl1* apices (Fig. 4A-B; S1 Dataset). However, only GRMZM2G093276, which encodes a ZIP zinc/iron transport protein, shows a significant increase in transcript levels in *lbl1* that is correlated with a decrease in siRNAs at the locus (S4 Dataset). Plants overexpressing ZIP proteins show a reduction in plant height and axillary bud outgrowth [39], [40], but such plants are otherwise morphologically normal. As such, a contribution of ZIP deregulation to the severe polarity defects of *lbl1* mutants seems speculative.

In addition, levels of eight miRNAs are significantly changed in *lbl1* (Fig. 4B; S1 Dataset). However, even though LBL1 is necessary for the proper spatiotemporal pattern of miR166 accumulation [17], its levels overall appear not significantly changed in the mutant. Unexpectedly, miR156, which represses the juvenile to adult phase transition [41], is upregulated in *lbl1*. *Arabidopsis* ta-siRNA biogenesis mutants exhibit an accelerated transition from the juvenile to the adult phase [2], [9], whereas increased levels of miR156 in *lbl1* might imply a delayed vegetative phase change. It is conceivable that rather than contributing to the phenotype, the changes in miR156 levels are a consequence of the *lbl1* phenotype. Similarly, while miR169, miR528, and miR529 are more abundant in *lbl1*, transcript levels for their targets remain unchanged in the mutant. The abaxialized leaf phenotype of *lbl1* can however not account for the increased accumulation of miR390, as this small RNA is expressed on the adaxial side of leaf primordia [42]. Instead, upregulation of miR390 in *lbl1* is consistent with feedback regulation in the *TAS3* ta-siRNA pathway [42] that is not expected to further impact the phenotype of *lbl1*.

The remaining loci differentially accumulating small RNAs in *lbl1* correspond primarily to DNA and retrotransposons (Fig. 4A-B; S1 Dataset). Interestingly, nearly all retrotransposons showing a significant change in 21- and 22-nt small RNA levels generate siRNAs preferentially or exclusively in the mutant (Fig. 4A-B). Moreover, more than 75% of these upregulated siRNA loci belong to the *gyma* class of Gypsy-like LTR retroelements (S1 Dataset), indicating a role for LBL1 specifically in the silencing of this class of retrotransposons. Recent studies in *Arabidopsis* revealed that ta-siRNA biogenesis components can act in a hierarchical manner to the transcriptional gene silencing pathway to silence repetitive regions in the genome [43]–[46]. While most repeats in the *Arabidopsis* genome are repressed at the transcriptional level through the action of PolIV/V, RDR2, and DCL3-dependent 24-nt heterochromatic siRNAs [47], in instances where this canonical silencing pathway is lacking or perturbed, 21- and 22-nt secondary siRNAs trigger the post-transcriptional repression of repetitive elements [43]–[46]. However, the observations presented here reveal an additional layer of small RNA-mediated transposon regulation. The data implies that when LBL1 activity is lost, a subset of *gyma* retroelements become targets for yet another small RNA pathway generating 21- and 22-nt LBL1-independent siRNAs. Unlike the repeat derived siRNAs generated by ta-siRNA pathway components in *Arabidopsis* [46], [48], the 21- and 22-nt LBL1-independent siRNAs map to the long terminal repeats (LTR) of the *gyma* elements. These seem to maintain the repression of these repeats, as *gyma* transcript levels are unchanged in the mutant.

Differentially expressed 24-nt small RNAs are also largely derived from retrotransposons and DNA transposons (Fig. 4A-B; S1 Dataset). For many of these repeats, siRNA levels are reduced in *lbl1*, consistent with the hypothesis that LBL1 also functions in the biogenesis of heterochromatic siRNAs associated with transcriptional gene silencing. While a role in RNA-dependent DNA methylation has been proposed for other distantly related members in the *Arabidopsis* SGS3-like protein family, SGS3 itself is not considered part of this subgroup [49]-[51]. Moreover, whether the SGS3-like proteins affect the accumulation of 24-nt siRNAs remains controversial. Consistent with a role for LBL1 in the production of 24-nt heterochromatic siRNAs, reduced expression of *lbl1* in extended transition stage leaves is correlated with demethylation and reactivation of *MuDR* transposons [52]. Importantly, the loci accumulating fewer 24-nt siRNAs in *lbl1* are distinct from the *gyma* retrotransposons generating increased levels of 21- and 22-nt small RNAs. This implies multiple contributions for LBL1 in the repression of repetitive elements in the genome: one via production of 24-nt siRNAs, and a distinct, not yet fully understood, role in the regulation of *gyma* retroelements. It also supports the presence of greater complexity in small RNA-mediated transposon silencing pathways, and that such alternate pathways may act preferentially on a select subset of transposon families [43], [45].

To assess the contribution of repeat-derived small RNAs to the *lbl1* defects, we next asked whether any non-phased, differentially expressed 21- or 22-nt small RNAs act in *trans* to regulate developmental genes at the post-transcriptional level in a manner analogous to miRNAs or ta-siRNAs. Assuming that all siRNAs are loaded into an AGO effector complex, we performed a target prediction and PARE analysis for those 21- and 22-nt differentially expressed small RNAs with three or more reads in either the wild-type or *lbl1* libraries. With a maximum target score of 4.5 [4], this identified eight genes targeted by the same *gyma*-derived siRNA (S3 Dataset). However, in contrast to the verified ta-siRNA targets (see above), these genes are not or scarcely expressed (<1 RPKM) in the vegetative apex and retain normal expression upon mutation of *lbl1*.

We further considered that the epigenetic regulation of transposable elements can influence expression of adjacent genes [53]. We, therefore, tested whether any of the repetitive or intergenic regions differentially accumulating 24-nt small RNAs are positioned in close proximity to a gene within the maize filtered or working gene sets. 106 of the 185 windows differentially accumulating 24-nt siRNAs are positioned within 10 kb of an annotated protein coding gene, but only three genes show a significant difference in transcript levels between wild-type and *lbl1* apices (S5 Dataset). GRMZM2G027495 and GRMZM2G016435 show increased expression in *lbl1,* even though 24-nt siRNAs levels at the adjacent repetitive and intergenic regions are also upregulated. This correlation is opposite to what is expected if silencing at the repeat region spreads into the adjacent gene, suggesting that expression of these genes is indirectly affected upon mutation of *lbl1*. The third gene, GRMZM2G089713 encodes for SHRUNKEN1. Its expression is significantly increased in *lbl1* consistent with a downregulation in 24-nt siRNAs at the adjacent repeat region. However, as SHRUNKEN1 modulates starch levels [54], a contribution to the developmental defects in *lbl1* is not immediately obvious.

Taken together, these analyses reveal unexpected crosstalk between small RNA pathways, with LBL1 making multiple unique contributions to the regulation of repeat-associated siRNAs, in addition to functioning in the biogenesis of ta-siRNAs. It is conceivable that, due to the highly repetitive nature of the maize genome, additional silencing pathways were co-opted to maintain genome integrity. The repeat-derived LBL1-dependent siRNAs are, however, unlikely to contribute substantially to the developmental defects of *lbl1* mutants. Instead, the data predicts that the essential role for *lbl1* in development reflects its requirement for the biogenesis of *TAS3*-derived tasiR-ARFs and the correct regulation of their *ARF3* targets.

### Mutations in *lbl1* and *rgd2* condition comparable adaxial-abaxial leaf polarity defects

**Figure 5:**
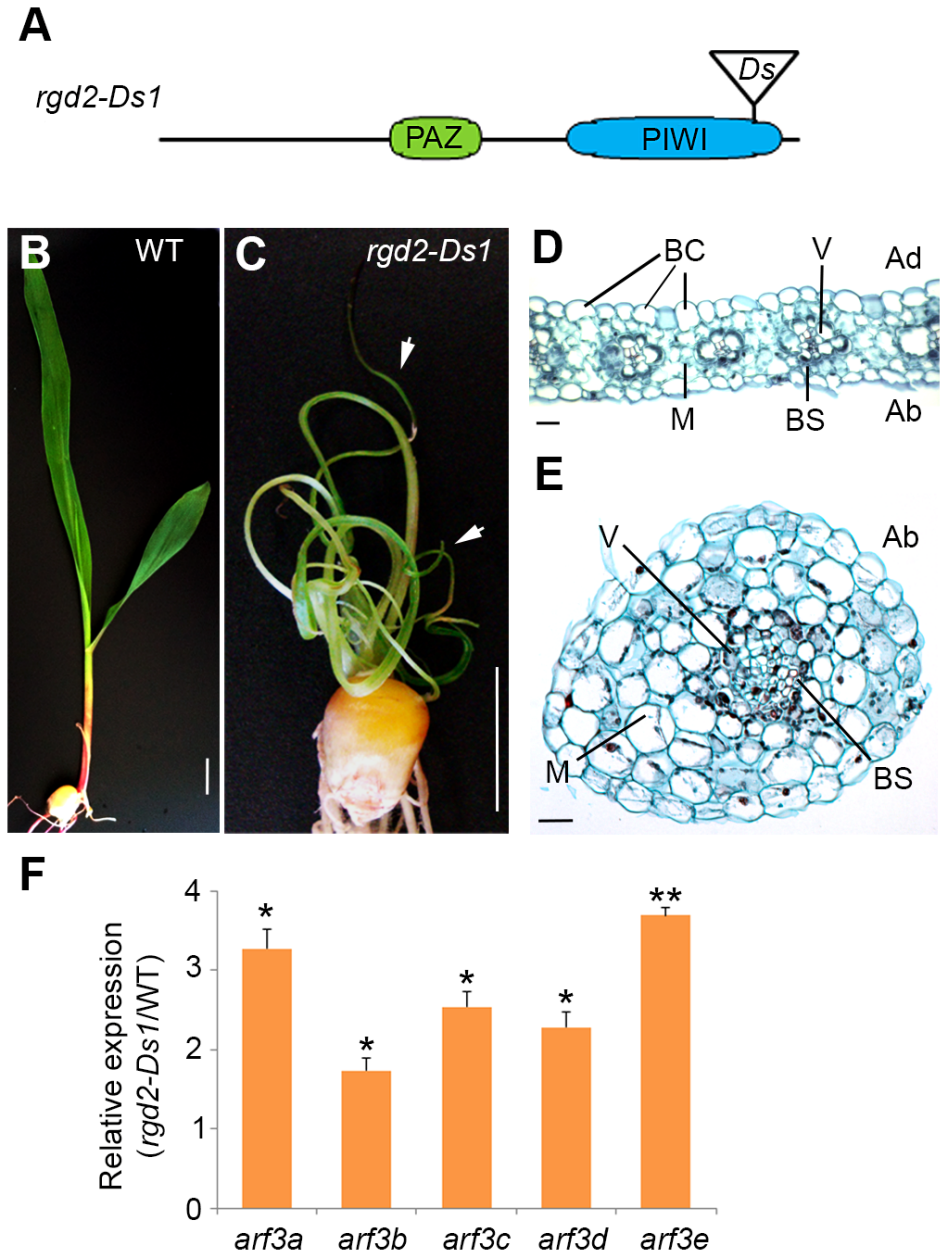
*rgd2-Ds1* mutants resemble the phenotype of *lbl1-rgd1* and develop radial abaxialized leaves. (A) *rgd2-Ds1* results from a *Ds* insertion into the essential PIWI domain of AGO7. (B, C) Compared to wild-type (B), *rgd2-Ds1* mutants (C) are severely stunted and develop very narrow and radial leaves (arrows). (D) Transverse section through a wild-type leaf blade. Bulliform cells mark the adaxial epidermis and within the vascular bundles, xylem differentiates adaxially to phloem. (E) Transverse section through a radial *rgd2-Ds1* mutant leaf. The mutant leaf consists of an irregular vascular cylinder surrounded by concentric rings of bundle sheath, mesophyll cells and abaxial epidermis, resembling those observed in *lbl1-rdg1* mutants [55]. (F) Expression levels for the *ARF3* genes are significantly increased in *rgd2-Ds1*. Transcript levels determined by qRT-PCR (mean ± SD; n = 3) normalized to wild-type are shown (* p<0.05, ** p-value<0.01). Ad, Adaxial; Ab, Abaxial; V, vein; BC, bulliform cells; BS, bundle sheath; M, mesophyll.

The finding that loss of tasiR-ARF activity and the correct regulation of its *ARF3* targets underlie the developmental defects of *lbl1* mutants is unexpected. Mutations in *rgd2* (*ago7*), which is likewise required for tasiR-ARF biogenesis, are reported to yield a phenotype distinct from *lbl1*. Plants homozygous for *rgd2-R* develop narrow strap-like leaves, but these maintain adaxial-abaxial polarity [18]. Based on this difference in phenotype, LBL1 was proposed to have functions other than in tasiR-ARF biogenesis that contribute to maize leaf polarity. The described *rgd2-R* allele, however, results from a transposon insertion in the first large intron of the gene, presenting the possibility that it is not a complete loss-of-function allele. Moreover, the *rgd2-R* phenotype has been characterized primarily in the Mo17 inbred background, and natural variation present between B73 and Mo17 is known to affect the phenotype of developmental mutants. We therefore introgressed the *rgd2-Ds1* allele (Fig. 5A), which contains a *Ds*-transposon insertion in the essential PIWI domain and is predicted to completely disrupt protein activity, into the B73 background. The phenotype of *rgd2-Ds1* in B73 is more severe than that described for *rgd2-R*, giving rise to seedlings with reduced thread-like leaves that often arrest shortly after germination (Fig. 5B-C). The thread-like *rgd2-Ds1* leaves lack marginal characters, including the saw tooth hairs and sclerenchyma cells, as well as a ligule, macrohairs, and bulliform cells that characterize the adaxial epidermis. In transverse sections, such leaves show a radial symmetry with photosynthetic and epidermal cells surrounding a central vascular bundle (Fig. 5D-E). These defects closely resemble the phenotype of *lbl1-rgd1* [17], [55], consistent with our finding that the developmental defects of *lbl1* reflect a loss of tasiR-ARF activity. In fact, the phenotype of the *rgd2-Ds1* null allele appears slightly more severe than that of *lbl1-rgd1*, and this is correlated with a more pronounced effect on *ARF3* expression. Transcript levels for *arf3a* and *arf3c-e* are significantly increased up to 3.5 fold in *rgd2-Ds1* seedling apices (Fig. 5F). In addition, expression of *arf3b* is changed significantly in *rgd2-Ds1,* albeit less than two-fold.

### Loss of tasiR-ARF mediated regulation of *ARF3* genes underlies the phenotype of ta-siRNA biogenesis mutants

**Figure 6:**
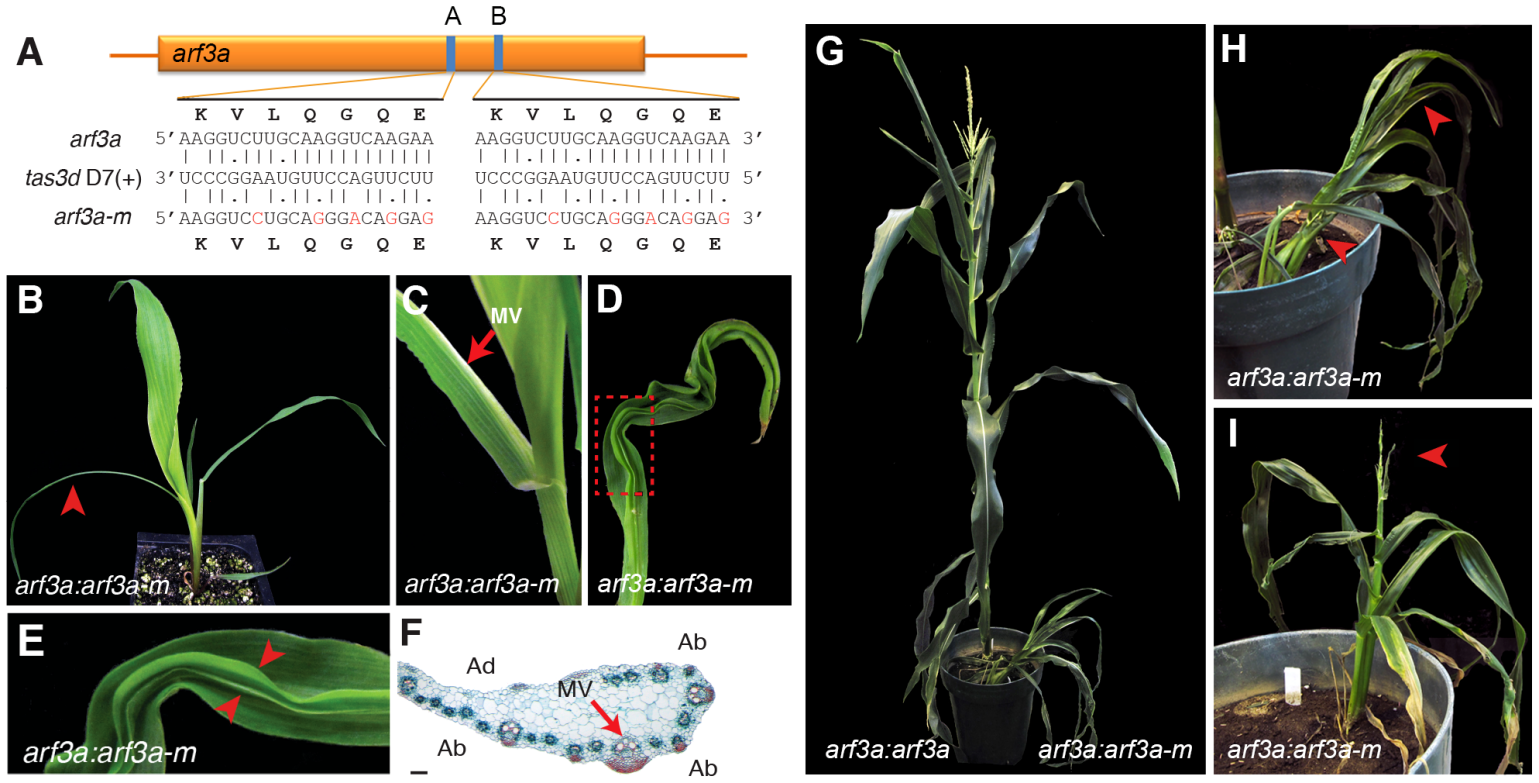
Expression of a tasiR-ARF insensitive *arf3a* transgene recapitulates the *lbl1* phenotype. (A) Mutations were introduced in both *arf3a* tasiR-ARF binding sites that reduce complementarity without affecting the amino acid sequence. Sequences of the tasiR-ARF target sites in *arf3a* and *arf3a-m* with base pairing to the *tas3d* D7(+) ta-siRNA, are shown. (B-E) Expression of *arf3a-m* from the *arf3a* regulatory regions produces a seedling phenotype with similarities to weak alleles of *lbl1* [55]. *arf3a:arf3a-m* seedlings develop thread-like abaxialized leaves (arrowhead in B) and half leaves (C), as well as leaves with ectopic blade outgrowths surrounding abaxialized sectors on the upper leaf surface radial (D, E). (F) Analogous to the half leaves present in weak *lbl1* mutants, half leaves on *arf3a:arf3a-m* transgenic plants show a partial radial arrangement of vascular bundles around the midvein (arrow). (G) Compared to the near wild-type phenotype of *arf3a:arf3a* transgenic plants (left) the stature of *arf3a:arf3a-m* plants (right) is severely reduced. (H-I) Such plants display defects in vegetative and reproductive development. Arrows in (H) mark half leaves, and in (I), a strongly reduced and sterile male inflorescence. Ad, Adaxial; Ab, Abaxial; MV, midvein. Scale bar = 100 µm.

The fact that mutations in *lbl1* and *rgd2* condition comparable adaxial-abaxial leaf polarity defects supports the finding that LBL1 contributes to development through the biogenesis of *TAS3*-derived ta-siRNAs. To confirm that this requirement lies specifically in the production of tasiR-ARFs and the downstream regulation of the *ARF3* abaxial determinants, we generated transgenic lines that express either a native or tasiR-ARF-resistant version of *arf3a*. The latter (*arf3a-m*) harbors silent mutations in each of the two tasiR-ARF target sites (Fig. 6A). Most plants expressing the native *arf3a* cDNA from the endogenous *arf3a* regulatory regions (*arf3a*:*arf3a*) are phenotypically normal. Only occasionally do such plants develop slightly narrower leaves and these become less evident as the plant matures. In contrast, transgenic plants expressing the tasiR-ARF insensitive *arf3a*:*arf3a-m* transgene displayed pronounced vegetative and reproductive abnormalities (Fig. 6B-I). *arf3a*:*arf3a-m* seedlings resemble seedlings homozygous for the weak *lbl1-ref* allele, and develop half leaves and thread-like abaxialized leaves, as well as leaves with ectopic blade outgrowths surrounding abaxialized sectors on the upper leaf surface [55]; (Fig. 6B-F). However, as such plants matured, their phenotypes became progressively more severe and resembled the phenotypes of *lbl1-rgd1*. Mature *arf3a*:*arf3a-m* plants have a dramatically reduced stature (Fig. 6G-H), and exhibit developmental abnormalities in both male and female inflorescences that result in complete sterility (Fig. 6I). Thus, misregulation of *arf3a* alone is sufficient to recapitulate phenotypic defects seen in *lbl1* and *rgd2* mutants [17], [18], [55]. The initial milder phenotypes of *arf3a*:*arf3a-m* plants are potentially explained by the fact that only *arf3a* expression is affected in these plants, as ARF3 has been shown to condition dose-dependent phenotypes in *Arabidopsis* [10]. These data demonstrate a conserved role for the ARF3 transcription factors in promoting abaxial fate, and confirm our findings from genome-wide analysis that LBL1 contributes to development through the biogenesis of *TAS3*-derived tasiR-ARFs and the regulation of their *ARF3* targets. Moreover, the severe defects conditioned by mutations in *lbl1* and *rgd2* thus underscore the importance of the tasiR-ARF *ARF3* regulatory module to maize development.

### Conclusions

The present work reveals substantial diversity in small RNA pathways across plant species, both in the regulation of repeat-associated siRNAs and the spectrum of phased siRNAs. Only *TAS* loci belonging to the *TAS3* family are active in the maize vegetative apex. In other plant species for which genome-wide small RNA analyses were completed, additional phased siRNA loci belonging to either the one-hit or two-hit sub-families were identified [21], [24], [26]–[28], [31], [36], [37], [56]. Some of the observed diversity may reflect variation in small RNA pathways across different tissue types, but our results indicate that essential steps in the one-hit phased siRNA pathway may function distinctly in the maize seedling apex. Whether this reflects a broader difference between monocots and dicots could be resolved by a similar in depth analyses of phased siRNAs in vegetative apices of e.g. rice and *Brachypodium*.

Our data further shows that loss of tasiR-ARF mediated regulation of *ARF3* genes is responsible for the developmental phenotypes of ta-siRNA biogenesis mutants in maize. Mutants affecting ta-siRNA biogenesis display phenotypes that differ widely from species to species. The *TAS3* ta-siRNA pathway, including the regulation of *ARF3* targets, is conserved throughout land plant evolution, but the population of phased siRNAs and their targets otherwise vary extensively [4], [5], [10]–[19]. Our findings indicate that this diversity in *TAS* pathways cannot fully account for the phenotypic differences of ta-siRNA biogenesis mutants. As in maize, the developmental defects of *Arabidopsis* and *Medicago* ta-siRNA biogenesis mutants can be mimicked by overexpression of a tasiR-ARF insensitive allele of *ARF3* [8]–[9], [14]. Nonetheless, in contrast to the severe polarity phenotype of *lbl1* leaves [17], *Arabidopsis* and *Medicago* ta-siRNA biogenesis mutants exhibit relatively subtle defects in leaf development, giving rise to downward-curled and highly lobed leaves, respectively [2], [12], [14], [57]. The fact that ARF3 proteins act as repressors of the auxin response [58] may be crucial to understanding these diverse phenotypes. Through their effect on the pattern and level of *ARF3* accumulation, the ta-siRNA pathway allows the auxin response to be modulated in a precise spatiotemporal manner. While the *TAS3* ta-siRNA pathway itself is highly conserved, its expression in time and space seems to vary across organisms. tasiR-ARFs act in the incipient maize leaf to polarize *ARF3* expression and establish adaxial-abaxial polarity, whereas tasiR-ARF biogenesis in *Arabidopsis* and *Medicago* is delayed until later in primordium development [10], [12], [14], [42]. Moreover, the nature and wiring of auxin responsive gene networks regulated by the ARF3 transcriptional repressor may vary between plants. Indeed, the polarity network in *Arabidopsis* and maize appears to be wired differently, as reflected in the distinct redundancies between polarity determinants in these species [10], [59]. As such, divergence in the gene networks downstream of the ARF3 transcription factors or the spatiotemporal pattern in which these tasiR-ARF targets act emerge as a testable hypotheses to explain the diverse contributions of the ta-siRNA pathway to development in maize, *Arabidopsis*, and possibly other plant species as well.

## Materials and Methods

### Small RNA library construction and sequencing

Families segregating the *lbl1-rgd1* allele [17] introgressed at least three times into B73 were grown in growth-chambers at 16 hour 28°C/light and 8 hour 24°C/dark cycles. Shoot apices including the meristem and up to 5 leaf primordia were dissected from 2 week-old plants in triplicate. Total RNA was prepared using the mirVana RNA isolation kit (Life Techologies), and 1ug per sample used to generate small RNA libraries using the small RNA-seq kit (Illumina). RNA 3′ and 5′ adapters were ligated in consecutive reactions with T4 RNA ligase. Ligated RNA fragments were primed with an adapter-specific RT primer and reverse transcribed with Superscipt II reverse transcriptase (Life Technologies) followed by eleven cycles of amplification with adapter specific primers. Resulting cDNA libraries were separated on a 6% TBE gel and library fragments with inserts of 18-25p excised. Recovered cDNA libraries were validated by QC on an Agilent Bioanalyzer HiSens DNA chip (Agilent Technologies Inc.) and were sequenced for 50 cycles on the Illumina GAIIx according to Illumina protocols with one sample per lane. The same RNA samples from two biological replicates were also used for RNA deep sequencing by Macrogen Inc, Korea.

### Small RNA alignments

Trimmed reads 18 to 26 nt in length were aligned to the maize B73 RefGen_v2 genome (release 5a.57) using Bowtie v0.12.7 [60]. While the observations presented in this study are robust across a wide range of mapping parameters, the specific data presented uses the following standard filtering criteria [28], [31]: only perfectly matched reads were considered and, taking into consideration the characteristics of previously described miRNA and ta-siRNA loci, a maximum of 20 alignments per read were reported. Reads matching known structural RNAs (rRNAs, tRNAs, sn-RNAs and sno-RNAs) from Rfam 10.0 [61] were removed from further analysis. As expected, considering only uniquely mapped reads eliminated several of the developmentally important ta-siRNAs and miRNAs. Whereas allowing 100 alignments per read in both the phased and LBL1-dependent siRNA analyses identified some additional and distinct repeat loci without impacting the overall conclusions.

### Small RNA differential abundance analysis

Using similar criteria as previously described [24], [62], the abundances of small RNA reads in each individual library were calculated using non-overlapping 500 nt windows. Any windows with fewer than 10 reads total, across all libraries were removed from further analysis. For the remaining windows, edgeR [63] was used to model the counts distribution using a negative binomial model with common dispersion estimate. Differentially expressed loci were defined as windows with at least a 2-fold difference in abundance between the wild-type and *lbl1* samples, and an adjusted P-value<0.05, corrected according to the method of Benjamini and Hochberg [64]. Differentially expressed 24-nt small RNAs derived from the *lbl1* introgression interval were excluded from further analysis. Differential accumulation of total reads in the 18-26 nt size classes between wild-type and *lbl1* were calculated using a two-tailed t-test, as the millions of reads sequenced in each size class would follow an approximately normal distribution.

### Phased small RNA cluster identification

Mapped reads from the three wild-type libraries were normalized by the number of genome-matched reads in the library and pooled into a single database. Reads matching the forward and reverse strand were merged, adjusting for the 2-nt 3′ overhangs generated by DICER processing. Using a sliding window of 500 bp, genomic regions containing at least 5 reads of 21-nt with a phasing distance of exactly 21-nt were identified as candidate phased clusters. To identify those candidate clusters in which the majority of small RNAs are phased, we modified the phasing score calculation of De Paoli et al. [65] to the following: 
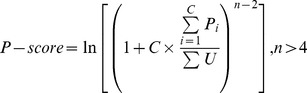
where n is the number of phase cycle positions occupied by at least one small RNA read, C is the number of phase cycles that fit within a 500 bp window (23 for 21nt small RNAs, 22 for 22-nt small RNAs, 20 for 24-nt small RNAs), P is the abundance of reads in each phase cycle, and U is the abundance of out of phase reads in the window. Analogous to previous studies [28], [31], a stringent threshold of P-score≥25 was used to identify phased siRNA clusters.

### Target prediction and PARE analysis

Target predictions for ta-siRNAs and *lbl1*-dependent siRNAs were made using Target Finder, allowing a maximum score = 4.5. Scoring was assigned as described previously [4]. PARE libraries were generated from B73 apex tissues as described previously [35]. PARE analysis was performed according to Zhai et al. [31], with the following minor modification: two windows flanking each predicted target site were defined and the total abundance of PARE tags matching to (1) a small window “W_S_” of 5 nt (cleavage site ±2 nt), and (2) a large window “W_L_” of 31 nt (cleavage site ±15 nt) calculated. Cleavage sites were filtered to retain only those for which W_S_/W_L_≥0.75 and W_S_≥4.

### RNAseq and differential expression analysis

Reads were trimmed and aligned to the B73 RefGen_v2 genome (release 5a.57) and the annotated exon junctions (version 5b_AGPv2) using TopHat v1.2.0. The majority (78–80%) of the trimmed reads were uniquely mapped and further analyzed for read counts per genes in the two replicates for each wild-type and *lbl1* mutant samples. RPKM values were determined using the Cufflinks package Cuffdiff v1.0.3 with default parameters, except that the “minimum alignment count” (-c) was set to 50 [66]. Differentially expressed transcripts were selected as those showing a 2-fold change in expression with a Cuffdiff determined, BH-corrected P-value<0.05. To analyze expression of repetitive elements, RNAseq reads were allowed to map up to 50 times to genome. Any multi-mapping reads were weighted 1/n, where n is the number of possible alignments. A counts table containing the total reads mapped to each repeat locus in each wild-type and *lbl1* replicate was analyzed as above.

### Generation of *arf3a*:*arf3a* and *arf3a*:*arf3a-m* transgenic lines

The MultiSite Gateway System (Invitrogen) was used to create the *arf3a:arf3a* and the *arf3a:m-arf3a* constructs. The coding sequences of *arf3a* (GRMZM2G030710_T01), and the *arf3a* regulatory regions (2.838 kb promoter and 1.059 kb 3′UTR) were amplified and cloned into pDONR entry vectors. To generate the tasiR-ARF insensitive *arf3a* version, mutations were introduced at the two tasiR-ARF target sites using megaprimer PCR mutagenesis. Entry clones for each of the two constructs were combined in a single MultiSite Gateway LR recombination reaction with the pTF101 Gateway-compatible maize transformation vector. Positive clones were transferred into *Agrobacterium tumefaciens* strain EHA101 and transformed into maize by the Iowa State University Plant Transformation Facility.

### Histological analysis

The *rgd2-Ds1* allele [18] was introgressed six times into B73, and segregating families were grown in growth-chambers at 16 hour 28°C/light and 8 hour 24°C/dark cycles. Leaves from mutant and wild-type plants were fixed and embedded as described [67]. Paraplast blocks were sectioned at a thickness of 10 µM and stained with Safranin-O and Fast Green according to Johansen's method.

### Quantitative RT-PCR analysis

Shoot apices were dissected from 12 day-old seedlings of *lbl1-rgd1*, *rgd2-Ds1,* and their respective non-mutant siblings. Total RNA was prepared using Trizol reagent (Invitrogen) and treated with DNase I (Promega). cDNA from 500 ng of RNA per sample was synthesized using Superscript III First-Strand Synthesis System (Invitrogen) according to manufacturer's protocol. Gene-specific primers were designed (sequence available upon request) for use with iQ SYBR Green Supermix (BioRad) in qPCR. The specificity of all amplification products was determined using dissociation curve analyses. Relative quantification (RQ) values were calculated based on at least three biological and two technical replicates using the 2^−ΔCt^ method, with the ΔCt of *glyceraldehyde-3-phosphate dehydrogenase* (*gapc*) as normalization control, taking into consideration the efficiencies of each primer pair as described [68], [69].

### Data access

High throughput sequencing data, both raw and processed files, has been submitted to the Gene Expression Omnibus and is available upon publication at accession number GSE50557.

## Supporting Information

S1 Figure *lbl1* shows a subtle effect on the overall small RNA population. The small RNA size distribution profiles are similar for wild-type and *lbl1*, but the 21-, 22-, and 24-nt small RNA levels (asterisks) differ significantly (p<0.05). Values shown are the mean normalized read counts (RPM) and SD from three independent biological replicates.
(TIF)

S2 Figure Informatics flowchart for the identification of phased and/or LBL1-dependent small RNAs. The 21-, 22-, and 24-nt small RNA reads that perfectly matched to the unmasked B73 reference genome served as inputs into two distinct informatics pipelines, with a maximum of 20 alignments per read analyzed. Such reads from wild-type and *lbl1* were analyzed for LBL1 regulated small RNAs using the filters outlined in the right arm of the flowchart. The genome-matched reads from wild-type samples were also used for the identification of phased siRNA clusters using the left arm of the flowchart. Those 21-nt phased siRNA clusters with a P-score≥25 that are downregulated in *lbl1* were considered potential new phased siRNA loci.
(TIF)

S3 Figure Genome-wide analysis of phased siRNA loci. (A) Phasing analysis identified 16, 102, and 8 loci that generate 21-, 22-, and 24-nt phased siRNAs, respectively. These correspond primarily to repetitive regions in the genome and their small RNA levels are mostly unchanged in *lbl1*. Phased siRNA levels at 8 low copy regions are changed significantly (q-value<0.05) in *lbl1* mutants. (B, C) Features of loci generating phased 22-nt small RNAs. (B) Phased 22-nt siRNAs map to the large intron of gene GRMZM2G181081, which is predicted to fold into a long hairpin RNA. (C) GRMZM2G481425 and GRMZM2G180676 generate overlapping antisense transcripts that give rise to phased 22-nt siRNAs.
(TIF)

S4 Figure Organization of *TAS3* loci generating phased 21-nt ta-siRNAs. (A-G) Distribution and abundance of small RNAs from *tas3a* (A), *tas3b* (B), *tas3d* (C), *tas3f* (D), *tas3g* (E), *tas3h* (F), and *tas3i* (G). The top graphs in each panel show the size distribution profiles for small RNAs at the respective *TAS* locus (mean normalized read counts ± SD; n = 3). The majority of ta-siRNAs are 21-nt long, except at *tas3f* and *tas3h*, which generate a relatively abundant class of 22-nt siRNAs. The middle graphs show the number of normalized reads (RPM) for ta-siRNAs in phase with the 3′ miR390 cleavage site, whereas the bottom graphs show the abundances of out-of-phase small RNAs at each locus. Red boxes, miR390 binding sites; green boxes tasiR-ARFs; vertical dashed lines, the 21-nt register.
(TIF)

S1 Table Summary of sequenced small RNA reads.
(DOCX)

S2 Table Summary of sequenced mRNA reads.
(DOCX)

Dataset S1 Annotation of LBL1 regulated 21-, 22-, and 24-nt small RNA windows.
(XLS)

Dataset S2 Annotation of 21-, 22-, and 24-nt phased siRNA clusters.
(XLSX)

Dataset S3 RNAseq and PARE analysis of predicted small RNA targets.
(XLSX)

Dataset S4 RNAseq analysis of genes accumulating LBL1 regulated small RNAs.
(XLS)

Dataset S5 RNAseq analysis of genes nearby windows accumulating 24-nt LBL1 regulated small RNAs.
(XLSX)
