# Supplementary figures and images for: Genome-Wide Analysis of leafbladeless1-Regulated and Phased Small RNAs Underscores the Importance of the TAS3 ta-siRNA Pathway to Maize Development
Source: PLoS Genet. 2014 Dec 11;10(12):e1004826. doi: 10.1371/journal.pgen.1004826 (PMC4263373; doi:10.1371/journal.pgen.1004826)

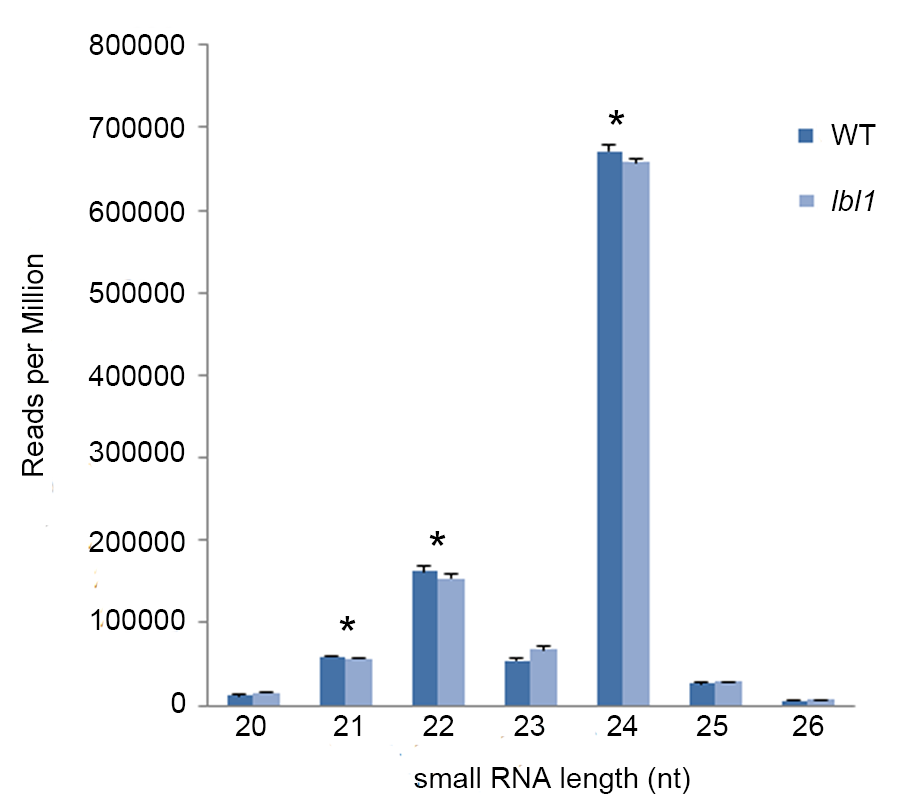

Supplement: S1 Figure — lbl1 shows a subtle effect on the overall small RNA population. The small RNA size distribution profiles are similar for wild-type and lbl1, but the 21-, 22-, and 24-nt small RNA levels (asterisks) differ significantly (p<0.05). Values shown are the mean normalized read counts (RPM) and SD from three independent biological replicates. (TIF) [file pgen.1004826.s001.tif]

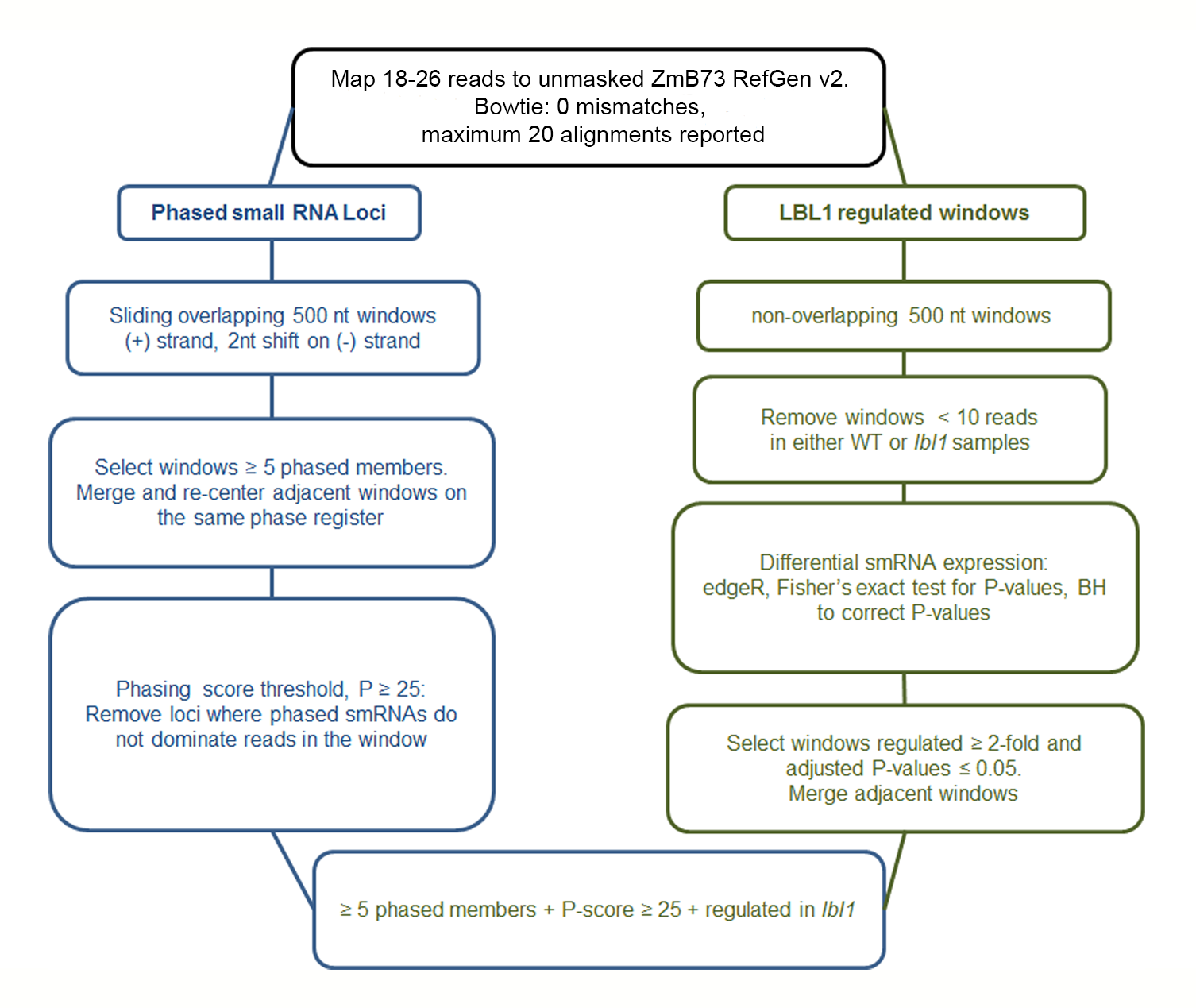

Supplement: S2 Figure — Informatics flowchart for the identification of phased and/or LBL1-dependent small RNAs. The 21-, 22-, and 24-nt small RNA reads that perfectly matched to the unmasked B73 reference genome served as inputs into two distinct informatics pipelines, with a maximum of 20 alignments per read analyzed. Such reads from wild-type and lbl1 were analyzed for LBL1 regulated small RNAs using the filters outlined in the right arm of the flowchart. The genome-matched reads from wild-type samples were also used for the identification of phased siRNA clusters using the left arm of the flowchart. Those 21-nt phased siRNA clusters with a P-score≥25 that are downregulated in lbl1 were considered potential new phased siRNA loci. (TIF) [file pgen.1004826.s002.tif]

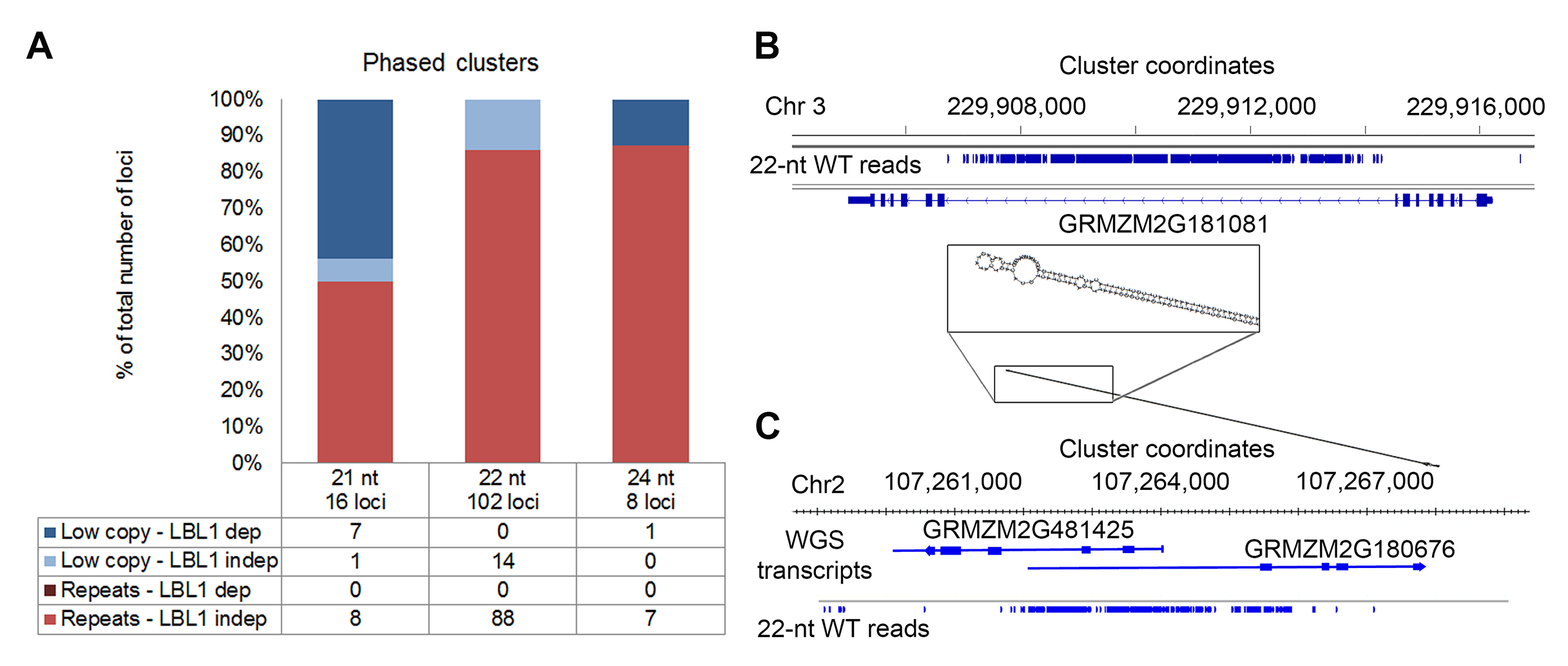

Supplement: S3 Figure — Genome-wide analysis of phased siRNA loci. (A) Phasing analysis identified 16, 102, and 8 loci that generate 21-, 22-, and 24-nt phased siRNAs, respectively. These correspond primarily to repetitive regions in the genome and their small RNA levels are mostly unchanged in lbl1. Phased siRNA levels at 8 low copy regions are changed significantly (q-value<0.05) in lbl1 mutants. (B, C) Features of loci generating phased 22-nt small RNAs. (B) Phased 22-nt siRNAs map to the large intron of gene GRMZM2G181081, which is predicted to fold into a long hairpin RNA. (C) GRMZM2G481425 and GRMZM2G180676 generate overlapping antisense transcripts that give rise to phased 22-nt siRNAs. (TIF) [file pgen.1004826.s003.tif]

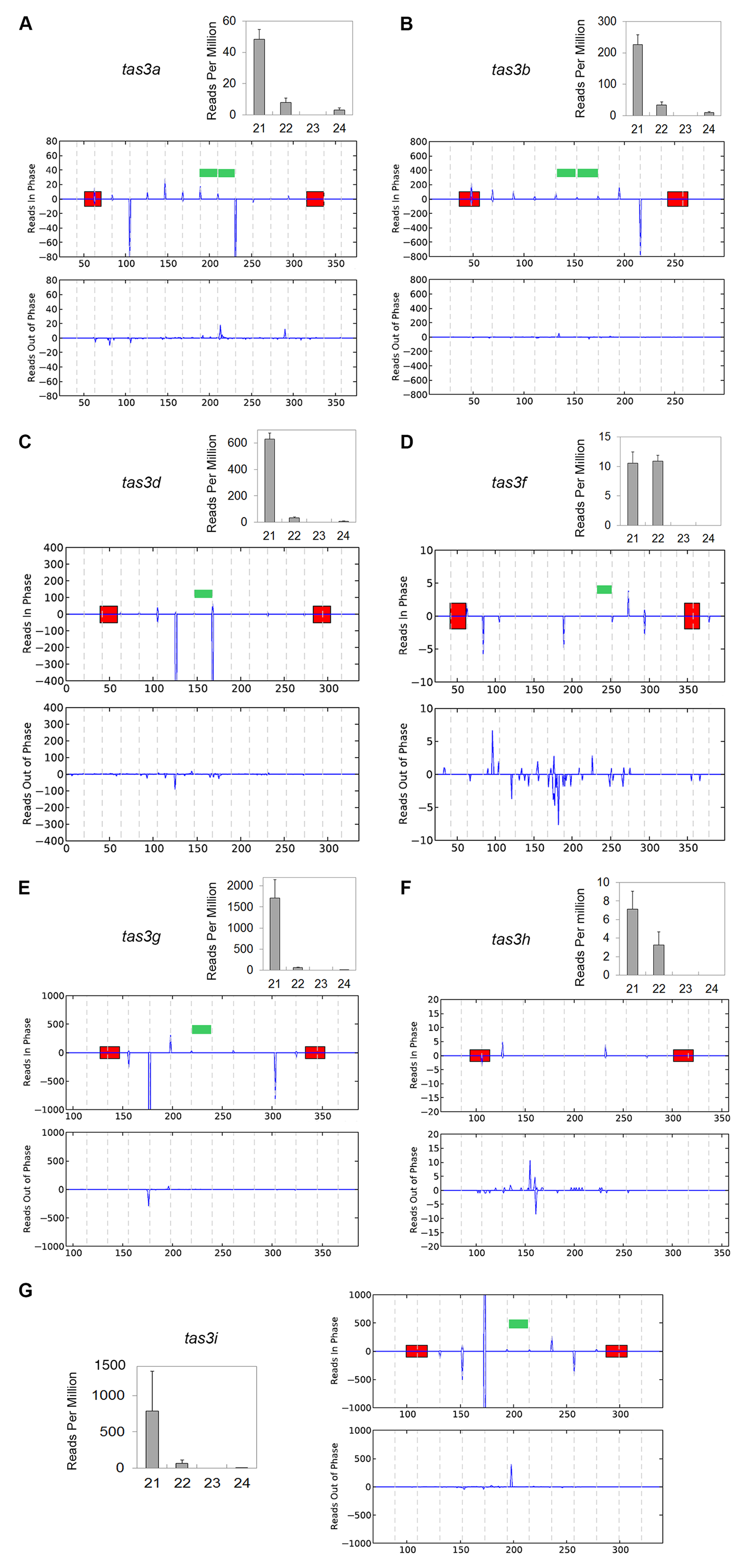

Supplement: S4 Figure — Organization of TAS3 loci generating phased 21-nt ta-siRNAs. (A-G) Distribution and abundance of small RNAs from tas3a (A), tas3b (B), tas3d (C), tas3f (D), tas3g (E), tas3h (F), and tas3i (G). The top graphs in each panel show the size distribution profiles for small RNAs at the respective TAS locus (mean normalized read counts ± SD; n = 3). The majority of ta-siRNAs are 21-nt long, except at tas3f and tas3h, which generate a relatively abundant class of 22-nt siRNAs. The middle graphs show the number of normalized reads (RPM) for ta-siRNAs in phase with the 3′ miR390 cleavage site, whereas the bottom graphs show the abundances of out-of-phase small RNAs at each locus. Red boxes, miR390 binding sites; green boxes tasiR-ARFs; vertical dashed lines, the 21-nt register. (TIF) [file pgen.1004826.s004.tif]
