# Supplementary material for: Genome-Wide Analysis of leafbladeless1-Regulated and Phased Small RNAs Underscores the Importance of the TAS3 ta-siRNA Pathway to Maize Development
Source: PLoS Genet. 2014 Dec 11;10(12):e1004826. doi: 10.1371/journal.pgen.1004826 (PMC4263373; doi:10.1371/journal.pgen.1004826)
Supplement: S1 Table — Summary of sequenced small RNA reads. (DOCX) [file pgen.1004826.s005.docx]

**Table S1: Summary of Sequenced small RNA Reads**

|  | **WT_1** | **WT_2** | **WT_3** | ***lbl1*_1** | ***lbl1*_2** | ***lbl1*_3** |
| --- | --- | --- | --- | --- | --- | --- |
| **18-26 nt Reads** | 3934045 | 4188485 | 3555224 | 3378193 | 3295362 | 3489183 |
| **Genome-Matched Reads** | 3710371 | 3872793 | 3305761 | 3076677 | 3000592 | 3187747 |
| **(% of Total Reads)** | (94.3%) | (92.5%) | (93.0%) | (91.1%) | (91.1%) | (91.4%) |
| **Distinct Genome-Matched Reads** | 1618249 | 1672721 | 1482263 | 1404666 | 1413360 | 1440737 |
| **(% of Genome-Matched Reads)** | (43.6%) | (43.2%) | (44.5%) | (45.6%) | (47.1%) | (45.2%) |
